# Infection with *Schistosoma mansoni* has an Effect on Quality of Life, but not on Physical Fitness in Schoolchildren in Mwanza Region, North-Western Tanzania: A Cross-Sectional Study

**DOI:** 10.1371/journal.pntd.0005257

**Published:** 2016-12-27

**Authors:** Safari Kinung’hi, Pascal Magnussen, Godfrey Kaatano, Annette Olsen

**Affiliations:** 1 National Institute for Medical Research, Mwanza Research Centre, Mwanza, Tanzania; 2 Parasitology and Aquatic Diseases, Faculty of Health and Medical Sciences, University of Copenhagen, Copenhagen, Denmark; 3 Centre for Medical Parasitology, Faculty of Health and Medical Sciences, University of Copenhagen, Copenhagen, Denmark; George Washington University School of Medicine and Health Sciences, UNITED STATES

## Abstract

**Background:**

Infection with *Schistosoma mansoni* negatively impact children’s physical health and may influence their general well-being. The aim of this study was to investigate the effect of *S*. *mansoni* infections on a panel of morbidity indicators with emphasis on quality of life (PedsQL; measured in four different dimensions) and physical fitness (measured as VO_2_ max) among 572 schoolchildren aged 7–8 years.

**Methodology/Principal findings:**

Prevalence of *S*. *mansoni* infections was 58.7%, with an arithmetic mean (95% CI) among positives of 207.3 (169.2–245.4) eggs per gram (epg). Most infections were light (56.5%), while 16.4% had heavy infections. Girls had significantly higher arithmetic mean intensities (95% CI) than boys (247.4 (189.2–305.6) vs. 153.2 (110.6–195.8); *P* = 0.004). A total of 30.1% were anaemic with no sex difference. Stunting and wasting was found in less than 10% of the population. There was no association between *S*. *mansoni* prevalence or intensities and the following parameters: anthropometry, anaemia, liver or spleen pathology in neither univariable nor multivariable linear regression analyses. However, in univariable analyses children with *S*. *mansoni* infection had a significantly lower score in emotional PedsQL (95% CI) than uninfected (77.3 (74.5–80.1) vs. 82.7 (79.9–85.5); *P* = 0.033) and infected children had a higher VO_2_ max (95% CI) compared to uninfected (51.4 (51.0–51.8) vs. 50.8 (50.3–51.3); *P* = 0.042). In multivariable linear regression analyses, age, *S*. *mansoni* infection, haemoglobin and VO_2_ max were significant predictors for emotional PedsQL while significant predictors for VO_2_ max were physical PedsQL, height, age and haemoglobin. *S*. *mansoni* infection was thus not retained in the multivariable regression analyses on VO_2_ max.

**Conclusions/Significance:**

Of the measured morbidity parameters, *S*. *mansoni* infection had a significant effect on the emotional dimension of quality of life, but not on physical fitness. If PedsQL should be a useful tool to measure schistosome related morbidity, more in depth studies are needed in order to refine the tool so it focuses more on aspects of quality of life that may be affected by schistosome infections.

## Author Summary

Millions of school-age children in Tanzania are infected with *Schistosoma mansoni* which have an impact on their physical health by reducing growth and fitness, causing anaemia and liver and spleen pathology. Apart from these measurable physical parameters, self-rating of well-being by administration of quality of life questionnaires has been proposed for measuring schistosome related morbidity. A total of 572 schoolchildren aged 7–8 years were examined for *S*. *mansoni* infection, anaemia, malnutrition and liver/spleen enlargement. Furthermore, the children participated in a 20 metres shuttle run fitness test and answered a quality of life questionnaire for children. While there was no association between *S*. *mansoni* prevalence or intensities and anaemia, malnutrition, fitness or organ enlargement, *S*. *mansoni* infected children had a significantly lower self-perceived emotional quality of life. Thus, infected children were more often scared, sad, angry or worried about their future compared to their uninfected peers. More in depth studies are needed in order to refine the questionnaire so it focuses more on aspects of quality of life that may be affected by schistosome infections.

## Introduction

Schistosomiasis is a serious neglected tropical disease and a major public health problem especially in Sub-Saharan Africa [1,2]. Of the 230 million estimated cases of schistosomiasis more than 80% occur in Sub-Saharan Africa, and the United Republic of Tanzania has the second highest number of cases in the region (19 million) only surpassed by Nigeria’s 29 million [2]. In Tanzania, both intestinal and urogenital schistosomiasis are endemic, but along the shores of Lake Victoria, the intestinal form caused by *S*. *mansoni* is especially abundant in school-age children, adolescents and in fishing communities [3,4].

The clinical consequences of *S*. *mansoni* infections result from tissue damage and blood loss caused by eggs trapped in host tissues. Chronic infection triggers host immune reactions which leads to granuloma formation in intestines, liver and spleen leading to hepatosplenic disease characterized by liver and spleen enlargement, portal hypertension and oesophageal varices [3,5]. Chronic infections particularly in children lead to marked non-specific symptoms such as anaemia, chronic inflammation, malnutrition, impaired growth, impaired mental development and general body weakness [1].

Ultrasonography (US) is the method of choice for detection and differentiation of liver pathology in intestinal schistosomiasis and pathology is positively related to duration and intensity of infection [6,7]. In Mwanza Region, US related morbidity was pronounced in adults with high percentages of periportal fibrosis (PPF), but enlargement of liver and spleen was not associated with neither infection or with the presence of PPF [8]. In a study of school and preschool children in the same area, only 2.3% showed PPF despite high rates of hepatomegaly and splenomegaly [9]. The explanation is probably that the evolution of PPF is believed to take at least 5 years [8,10] and therefore not common in young children. On the other hand, in acute infections, the left liver lobe enlarge, while in advanced disease the left liver lobe is normal or even reduced in size [8]. Ultrasonography therefore seems to be less adequate for evaluation of morbidity due to *S*. *mansoni* in young children and alternative methods could be useful. Physical fitness and perceived quality of life could be such methods. A number of studies have used the 20 metres shuttle run fitness test (20mSRT) for evaluating the effect of infection on physical fitness [11–14], but none of the studies found an association between the 20mSRT and schistosome infections.

Jia and colleagues [15,16] used the European quality of life questionnaire (EQ-5D plus) in populations in China with chronic and advanced *S*. *japonicum* infections, and in Côte d’Ivoire another questionnaire (SF-36v2) was used to assess the physical burden of polyparasitism [17]. Currently, only two studies have quantified the effect of schistosome infection on overall wellbeing or quality of life using the Pediatric Quality of Life Inventory (PedsQL) [18,19]. Both studies used the same short form of the tool as the present study (PedsQL 4.0 SF15) and both found a lack of association between quality of life and infections at the individual level.

The aim of this study was to investigate the effect of *S*. *mansoni* infection on a panel of morbidity indicators with emphasis on physical fitness and quality of life among schoolchildren in Mwanza Region, Tanzania.

## Materials and Methods

### Ethics statement on subject recruitment

The study was reviewed and approved by the Medical Research Coordination Committee (MRCC) of the National Institute for Medical Research (NIMR), Tanzania (ethics clearance certificate no. NIMR/HQ/R.8a/Vol.IX/1022) and the University of Georgia Institutional Review Boards, Athens, Georgia (2011-10353-1). Only children who assented to participate and had parental or guardian written informed consent were eligible for inclusion.

Before examination and sample collection the reason for the survey and the procedure of sample collection were explained to the children and the adult population in the communities including local leaders, school administration, teachers and health and education personnel. Each included person was assigned an identification number and results entered in a confidential file. Included communities were treated with a standard oral dose of praziquantel (40 mg/kg body weight) using a standardized dose pole [20]. The trial was registered with ClinicalTrials.gov (NCT02162875).

### Study area and population

This baseline study is the Tanzanian part of the SCORE (Schistosomiasis Consortium for Operational Research and Evaluation) project, and is a larger six-armed randomized controlled four years intervention trial with 25 villages in each arm previously described in detail elsewhere [4]. In short, the study was carried out from August to November 2011 in villages/schools within a 10 km distance from Lake Victoria in Mwanza Region of Tanzania where transmission of *S*. *mansoni* is perennial and where a prior site selection survey had identified a prevalence of *S*. *mansoni* of 25% or more. At commencement of the study, the region had 1,139 primary schools enrolling a total of 902,367 schoolchildren which is more than 95% of all school age children in the region [21]. In two of the six arms, four villages in each arm were randomly selected (but see restrictions below) to nest a cohort of 100 first-year students (aged 7–8 years) for morbidity assessments. Thus, 800 children were included at baseline. Proper sample size calculations were not done for the cohort study, but we enrolled the largest sample possible given the available resources.

The two arms with a total of 50 villages were situated in Geita, Sengerema, Misungwi, Nyamagana, Ilemena and Magu districts. For convenience, Geita and Magu districts were excluded and the selection of the eight villages was performed in the four districts closest to Mwanza. This reduced the total number of villages to 25 from which the eight villages were randomly selected (Trial protocol is attached in the supporting information files). In these villages (Bukumbi, Chamabanda, Isamilo, Kafundikile, Kasomeko, Katunguru, Nyamatongo, Nyamazugo) morbidity indicators were measured in addition to the parasitological investigations. These included: height, weight, haemoglobin (Hb), US detected organ pathology, physical fitness and quality of life. Baseline results from this cohort are presented.

### Parasitological investigations

Participants were given stool containers and asked to provide one fresh stool specimens on three consecutive days. A temporary field laboratory was set up in each of the visited schools and duplicate Kato-Katz thick smears using a 41.7 mg template [22] were processed from each specimen. The slides were transported to the National Institute for Medical Research and examined for *S*. *mansoni* eggs. Infections with soil-transmitted helminths (STHs) were not investigated as *Ascaris lumbricoides* and *Trichuris trichiura* are uncommon in this area [23,24] and eggs of hookworms would not be visible at the time of reading.

Number of eggs were multiplied by 24 and expressed as eggs per gram of stool (epg) and the mean from the total number of slides per person was calculated. Intensities were reported as geometric means and arithmetic means of epg among positive individuals only and as intensity groups according to WHO guidelines [25]; light (1–99 epg), moderate (100–399 epg) and heavy (≥400 epg).

### Anaemia assessment

Finger-prick blood samples were taken and Hb measured using a portable HemoCue photometer. Anaemia was defined as Hb values below 115 g/L, while severe anaemia was Hb values below 80g/L according to the World Health Organization guidelines [26].

### Anthropometric measurements

Height was measured on barefooted children using a locally made stadiometer. The child stood on the base of the stadiometer with heels, buttocks, shoulder blades and back of the head touching the vertical backboard and looking straight ahead. When correctly positioned, the ruler was lowered and the height measured in centimetres with one decimal. Weight was measured on a digital scale (TANITA Cooperation, Tokyo, Japan) on barefooted children having removed any excess clothing. The weight was measured in kilograms with one decimal. Height and weight were measured twice by the same examiner and the mean recorded. Z-scores were calculated using the Growth Reference Data tables for 5–19 year old children [27]. As the exact birthday of the children was not known, the Z-score limits of children aged 7 years and 6 months were used for the included 7 years old children and the Z-score limits of children aged 8 years and 6 months for the 8 years old children. Wasting was defined as a BMI-for-age Z-score of <-2 SD and stunting as a height-for-age Z-score <-2 SD.

### Abdominal ultrasonography

Abdominal US was performed using a portable ultrasound machine (Aloka Sonocamera SSD-500 with a 3.5 MHz curvilinear probe). Power was supplied from a portable generator. The examinations were performed according to the Niamey protocol [28] by a senior sonographer with extensive experience in field US of *S*. *mansoni* infected individuals. Children were examined lying on their backs with their legs stretched on an examination table. Measurements involved the length of the left liver lobe (mm), spleen length (mm), portal branch thickening and portal-vein diameter (PVD in mm). The liver texture was translated into six texture patterns, A-F_,_ as described in the Niamey protocol. Image patterns A and B were considered normal. Image pattern C and D were considered mild and moderate fibrosis, respectively, while liver pattern E and F were considered advanced PPF. Hepatomegaly (enlarged liver), splenomegaly (enlarged spleen) and increased PVD were defined as 2 standard deviations (SDs) above standard reference measurements for healthy uninfected children in corresponding height groups [28,29].

### Physical fitness

The 20mSRT was used to assess fitness. During the 20mSRT, children run continuously between two lines 20 meters apart, turning when signalled to do so by recorded beeps. A “shuttle” is defined as a run from one line to the other. The 20mSRT consists of 20 levels, each a minute long. At level one, the beeps are far apart with fewer shuttles than in later levels. As levels increase, the beeps get closer requiring the children to run faster and faster to reach the opposite line before the next beep. The running field was prepared on a flat surface in the school compound; the test was explained to the children and before the real test, three to five children not included in the cohort did a pilot run. Runners were separated at least one meter apart. Recorders were placed at each end, every recorder responsible for taking notes of three children. The recorder noted the level at which the test subject stopped and how many shuttles the child completed within that level e.g. if the subject starts level 4 and only completes two of the shuttles, the recorder will note “4” as the highest level reached and “2” as the number of shuttles completed at the highest level. These numbers are correlated to a maximal oxygen uptake, the VO_2_ max in mL/kg/min [12].

### Quality of life

Evaluation of quality of life was performed using the validated Pediatric Quality of Life Inventory 4.0 Short Form 15 (PedsQL4.0 SF15) instrument for children. The questionnaire consists of 15 questions and is divided in four parts with 3–5 questions in each. The four parts describe four dimensions of functioning: Problems with physical activities (physical), problems with feelings (emotional), problems with getting along with others (social) and problems with keeping up in school (school). The answers were scored from 0 to 4, where 0 is never, 1 is almost never, 2 is sometimes, 3 is often and 4 is almost always. Responses are transformed to scores: 100, 75, 50, 25 and 0, respectively, resulting in a scale range from 0–100 with the higher score indicating a perceived better quality of life.

### Statistics and data analysis

Data were analysed using IBM statistics SPSS version 21 (IBM, Armonk, NY). The χ^2^ test was used to test differences in proportions, the *t*-test was used to assess differences in normally distributed means and the Mann-Whitney test used for means, which were not found normally distributed. Linear multivariable regression analysis using a stepwise strategy (criteria for enter ≤0.05 and for remove ≥0.10) investigated factors significantly associated with the emotional PedsQL and VO_2_ max. All tests used the 5% level of significance.

## Results

One village (Kafundikile; n = 79, 52 boys and 27 girls) had to be excluded due to poor cooperation from the community for unknown reasons. In the remaining seven villages, 572 children aged 7–8 years participated in the survey and had complete parasitological and hematologic data of whom 315 (55.1%) were girls. Table 1 shows the included children for different parameters and the measured parameters by sex. Boys were significantly taller and heavier than girls.

**Table 1:** Baseline characteristics of children included in survey. *P*-values between sexes are shown.

Variable	All	Boys	Girls	*P*-values
	n = 572	n = 257, 44.9%	n = 315, 55.1%	
Age, years (range)	7.4 (7–8)	7.5 (7–8)	7.3 (7–8)	
*S*. *mansoni* positives, n % (95% CI)	336	143	193	0.17 ^a^
	58.7 (54.6–62.8)	55.6 (49.5–61.7)	61.3 (56.0–66.6)	
Arithmetic mean of positives epg (95%CI)	207.3 (169.2–245.4)	153.2 (110.6–195.8)	247.4 (189.2–305.6)	0.004 ^b^
Light, % (95% CI)	56.5 (51.2–61.8)	66.4 (58.6–74.2)	49.2 (42.1–56.3)	0.002 ^a^
Mod. & heavy, % (95% CI)	43.5 (38.2–48.8)	33.6 (25.8–41.4)	50.8 (43.7–57.9)	
Geometric mean of positives epg (95% CI)	66.5 (55.9–79.2)	49.8 (38.4–64.6)	82.4 (65.2–104.1)	0.005 ^c^
Anaemia prevalence, n % (95% CI)	172	74	98	0.55 ^a^
	30.1 (26.4–33.8)	28.8 (23.3–34.3)	31.1 (26.0–36.2)	
Hb level in g/L (95%CI)	120.0 (118.8–121.1)	120.3 (118.6–122.0)	119.7 (118.1–121.3)	0.62 ^c^
	n = 566	n = 255, 45.1%	n = 311, 54.9%	
Anthropometry				
Height in cm (95% CI)	123.3 (122.8–123.8)	124.4 (123.6–125.2)	122.3 (121.6–123.0)	<0.0005 ^c^
Weight in kg (95% CI)	22.3 (22.0–22.6)	23.0 (22.6–23.4)	21.8 (21.5–22.1)	<0.0005 ^c^
Stunting, % (95% CI)	7.5 (5.3–9.7)	8.9 (5.4–12.4)	6.3 (3.6–9.0)	0.24 ^a^
Wasting, % (95% CI)	8.9 (6.5-11-3)	10.5 (6.8–14.2)	7.6 (4.7–10.5)	0.23 ^a^
Ultrasound findings				
Liver pattern A	559 (98.8)	251 (98.4)	308 (99.0)	
Liver pattern B	3 (0.5)	2 (0.8)	1 (0.3)	
Liver pattern ≥ C	4 (0.7)	2 (0.8)	2 (0.6)	
Enlargement of left liver lobe, % (95% CI)	73.2 (69.5–76.9)	73.2 (67.7–78.7)	73.2 (68.3–78.1)	0.99 ^a^
Enlargement of spleen, % (95% CI)	28.3 (24.6–32.0)	31.4 (25.7–37.1)	25.7 (20.8–30.6)	0.14 ^a^
Enlargement of liver & spleen, %, (95% CI)	20.5 (17.2–23.8)	23.0 (17.7–28.3)	18.4 (14.1–22.7)	0.18 ^a^
Increased portal vein diameter, %, (95% CI)	7.8 (5.6–10.0)	8.3 (5.0–11.6)	7.4 (4.5–10.3)	0.68 ^a^
	n = 511	n = 225, 44.0%	n = 286, 56.0%	
Shuttle run (VO_2_ max) (mL/kg/min) (95% CI)	51.2 (50.9–51.5)	51.5 (51.0–52.0)	51.0 (50.6–51.4)	0.11 ^c^
	n = 446	n = 199, 44.6%	n = 247, 55.4%	
Quality of life, mean (95% CI)				
Physical PedsQL	84.2 (81.8–86.6)	81.8 (77.9–85.7)	86.1 (83.2–89.0)	0.11 ^b^
Emotional PedsQL	79.5 (77.5–81.5)	80.7 (77.8–83.6)	78.6 (75.9–81.3)	0.27 ^b^
Social PedsQL	84.3 (82.2–86.4)	82.8 (79.5–86.1)	85.4 (82.6–88.2)	0.24 ^b^
School PedsQL	80.9 (78.7–83.1)	79.8 (76.5–83.1)	81.8 (78.9–84.7)	0.39 ^b^
Total PedsQL	82.3 (80.5–84.1)	81.5 (78.7–84.3)	83.0 (80.6–85.4)	0.66 ^b^

^a^ Chi-square test

^b^ Mann-Whitney test

^c^ *t*-test.

### Prevalence of intestinal schistosomiasis and anaemia

Overall 336 children (58.7%) had *S*. *mansoni* infection with no sex differences, but girls had significantly higher *S*. *mansoni* infection intensities than boys (Table 1). More than half of the infected children (56.5%) had light infections; but 11.2% of the boys and 20.2% of the girls had heavy infections. A total of 30.1% were anaemic and there were no sex differences (Table 1). Of the 172 children with anaemia, only 7 children (4.1%) had severe anaemia. The prevalence and intensities of infection differed significantly among villages (*P* <0.0005). The lowest and highest prevalence recorded was 32.0% and 94.6%, respectively, while the lowest and highest arithmetic mean intensity among positive individuals were 41.2 and 423.1 epg, respectively.

Prevalence of anaemia varied from 24.0% to 40.9% while Hb levels ranged from 117.5 g/L to 122.7 g/L, but there were no significant differences in Hb levels and prevalence of anaemia between villages. There was no association between the prevalence or intensity of *S*. *mansoni* infection and the prevalence of anaemia or Hb level.

### Anthropometry and ultrasonography

There was no association between *S*. *mansoni* prevalence or intensities and height, weight, stunting or wasting, but stunting was significantly associated with anaemia. Thus, anaemia (95% CI) was present in 48.8% (33.7–63.9) of the stunted pupils compared to 30.1% (26.4–33.8) in the overall population (*P* = 0.005).

The liver could be palpated in only one child. Table 1 shows the percentage of children with enlarged left liver lobe, enlarged spleen, enlarged liver and spleen, and increased portal vein diameter. There were no sex differences in these parameters. Almost all children (99.3%) had liver image pattern A and B, while three children had liver image pattern C and only one child had liver image pattern F. There was no association between any of these parameters and *S*. *mansoni* infection.

### Physical fitness

Complete physical fitness data assessed by the 20mSRT were available for 511 children (90%). Sixty one children did not participate in the physical fitness test due to: sickness (5) absenteeism (24) and unknown (32). There was no difference between boys and girls in overall mean VO_2_ max (Table 1). Physical fitness was associated with *S*. *mansoni* infection (Table 2) but was neither associated with stunting nor wasting (S1 Data).

**Table 2:** Mean physical fitness measured as VO_2_ max of the shuttle run test and quality of life measured as physical, emotional, social, school and total PedsQL (measured on a 1–100 scale) and 95% confidence intervals (95% CI) by *S*. *mansoni* infection.

	*S*. *mansoni* infection		*P*-values
	Positive Mean (95% CI)	Negative Mean (95% CI)	
Shuttle run test; VO_2_ max	51.4 (51.0–51.8)	50.8 (50.3–51.3)	0.042 ^a^
Physical PedsQL	83.7 (80.7–86.7)	84.8 (81.0–88.6)	0.51 ^b^
Emotional PedsQL	77.3 (74.5–80.1)	82.7 (79.9–85.5)	0.033 ^b^
Social PedsQL	83.3 (80.5–86.1)	85.7 (82.4–89.0)	0.30 ^b^
School PedsQL	80.4 (77.5–83.3)	81.5 (78.1–84.9)	0.70 ^b^
Total PedsQL	81.4 (78.9–83.9)	83.6 (80.9–86.3)	0.47 ^b^

^a^ *t-*test

^b^ Mann-Whitney test.

In multivariable linear regression analysis including demographic, anthropometric, hematologic and parasitological parameters together with ultrasound and quality of life results, only age, height, physical PedsQL and Hb were identified as significant predictors of physical fitness (VO_2_ max) (Table 3). The regression coefficient of age corresponded to a 1.67 decrease in maximal oxygen uptake (mL/kg/min) for one year increase in age (from 7 to 8 years of age). The regression coefficient of height corresponded to a 0.10 increase in maximal oxygen uptake for every 1 cm increase in height. The regression coefficient of physical PedsQL corresponded to a 0.04 decrease in maximal oxygen uptake (mL/kg/min) for every 1 increase in score of quality of life. Finally, the regression coefficient of Hb corresponded to a 0.28 increase in maximal oxygen uptake for every increase in Hb with 1 g/L. Neither *S*. *mansoni* prevalence nor intensity of infection was predictors for VO_2_ max.

**Table 3:** Regression coefficients (*B*), 95% confidence intervals (95% CI) and corresponding *P* values of variables found to be significant predictors of fitness measured as VO_2_ max (mL/kg/min) in children ages 7–8 years in a multivariable linear regression model.

Variable	*B* (95%CI)	*P*-value
Age in years	-1.67 (-2.34, -1.00)	<0.0005
Height in cm	0.10 (0.05, 0.15)	<0.0005
Physical PedsQL^a^	-0.04 (-0.05,-0.03)	<0.0005
Haemoglobin in g/L	0.28 (0.06, 0.50)	0.013

n = 399, adjusted *R*^2^ = 0.17, overall *P*-value <0.0005

^a^ Measured on a 0–100 scale.

### Quality of life (PedsQL)

A total of 446 children responded to the questionnaire on quality of life (PedsQL). Floor effects (answers in the extreme low scaling range) of all 4 dimensions were between 0 and 0.2%, while the ceiling effect (answers in the extreme high scaling range) was all above 15% (20.8–46.7%). Floor and ceiling effects of <15% are considered acceptable, while those >15% are considered to provide less precise estimates [18]. Table 2 shows that only the emotional dimension was significantly associated with infection as infected individuals had a lower mean score of 5.4 compared to the uninfected ones. Thus infected pupils were more often scared, sad, angry or worried about their future compared to their uninfected peers. In a linear multivariable regression model including demographic, hematologic and parasitological parameters together with ultrasound and fitness results, only age, *S*. *mansoni* infections, Hb and VO_2_ max were significant predictors of emotional PedsQL (Table 4). The coefficient of 7.60 for age means that 8 year old children on average had a 7.60 higher score of emotional PedsQL than their 7 year old peers. The coefficient of -5.38 for *S*. *mansoni* infection means that infected children on average had a 5.38 lower score of emotional PedsQL than their uninfected peers. The regression coefficient of Hb corresponded to a 1.71 increase in score for every increase in Hb level with 1 g/L. Finally, the regression coefficient of VO_2_ max corresponded to a 0.70 decrease in emotional PedsQL score for every increase in VO_2_ max with 1 mL/kg/min.

**Table 4:** Regression coefficients (*B*), 95% confidence intervals (95% CI) and corresponding *P* values of variables found to be significant predictors of emotional PedsQL (measured on a 1–100 scale) in children ages 7–8 years in a multivariable linear regression model.

Variable	*B* (95%CI)	*P*-value
Age in years	7.60 (3.38, 11.82)	<0.0005
*S*. *mansoni* infection ^a^	-5.38 (-9.59, -1.18)	0.012
Haemoglobin in g/L	1.71 (0.25, 3.17)	0.022
Fitness as VO_2_ max ^b^	-0.70 (-1.31, -0.09)	0.025

n = 399, adjusted *R*^2^ = 0.07, overall *P*-value <0.0005.

^a^ Coded as not infected = 0 and infected = 1.

^b^ Measured in mL/kg/min.

## Discussion

The present study showed that of all the morbidity parameters measured only the emotional part of the self-perceived quality of life was significantly associated with *S*. *mansoni* infection in children aged 7–8 years. This is in contrast to the findings of the only two other studies which have aimed to quantify the effect of schistosomiasis infection on overall wellbeing or quality of life at the individual level using the same tools as was used in the present study.

Terer and co-workers [18] evaluated the PedsQL 4.0 SF15 in 5–18 year old children in a *S*. *haematobium* endemic area in five villages in Kenya and found that the total health-related quality of life (HrQoL) was significantly lower in villages with high prevalence of infection and among the lower socio-economic quartiles. However, *S*. *haematobium* egg positivity was significantly associated with reduced HrQoL in moderate prevalence villages but with increased HrQoL in high prevalence villages. Thus, there was no significant difference in any dimension of the questionnaire when they compared egg-positive and egg-negative children.

Hürlemann and co-workers [19] tested the PedsQL in almost 5,000 children aged 5–16 years in an area with *S*. *mansoni* and *S*. *haematobium* in Côte d’Ivoire. Prevalence was 3.7% and 5.7%, respectively, but malaria was highly endemic. There was no significant negative association between HrQoL and any parasite infections, but anaemia reduced self-rated HrQoL.

The emotional dimension of the PedsQL asks the following question: In the past one month, how much have you felt afraid or scared, sad or blue, angry or worried about what will happen to you? The possible answers were: never, almost never, sometimes, often or almost always. A score of 100 means never, while a score of 75 means almost never. As infected pupils had a mean score of 77.3 and uninfected ones 82.7, the mean for both groups was in the high end of the quality of life scale. It is difficult to compare the results of the present study with the study from Côte d’Ivoire [19] because the questionnaire was not exactly similar, but the Kenyan study [18] used the same questionnaire and found a smaller score (approximately between 60 and 70) of the emotional PedsQL than the present study. Apart from the obvious reasons that the Kenyan study investigated *S*. *haematobium*, which can result in visible symptoms, it could also be due to the fact that the study included teenagers which might have more serious emotional problems compared to the children between 7 and 8 years in the present study. It would have been interesting to see the results from the Kenyan children (5–18 years of age) divided into age groups as this may have made the results from Kenya and Tanzania more comparable. A study on the assessment of age-specific disability of chronic schistosomiasis in China included all age groups above 5 years, and it showed expectedly that disability increased with age as chronicity also increases with age, meaning that children between 5 and 14 years had significantly lower levels of disability than older age groups [15]. However, in this young age group 6.5% reported moderate or extreme problems related to anxiety or depression.

Another study from Côte d’Ivoire found no relationship between self-reported quality of life and any parasitic infections (*S*. *haematobium*, *S*. *mansoni*, hookworm, *Ascaris lumbricoides* and *Plasmodium* spp) [17], but this questionnaire focused on physical functioning and did not include an emotional dimension. Another Chinese study investigated the quality of life in patients with advanced schistosomiasis, but this only included patients above 30 years of age and is not comparable to the present study [16].

We found no effect of *S*. *mansoni* infection on physical fitness using the 20mSRT. This is in line with other studies using the same test. Recently Bustinduy and colleagues [11] concluded that the 20mSRT was a feasible test in an area in Kenya endemic for *S*. *haematobium*, *P*. *falciparum*, lymphatic filariasis, hookworm and *Trichuris trichiura* although none of the investigated infections were associated with fitness. The study included 1950 children (5–18 years of age), but sampled only one stool, urine and blood sample. Only age, anaemia, stunting and wasting (in males) were significant in a model stratified by sex. Müller and co-workers [12] investigated the effect of single, dual, triple, quadruple and quintuple infections on 20mSRT in 156 children (aged 7–15 years). The infections were *S*. *haematobium*, *P*. *falciparum*, *S*. *mansoni*, hookworm and *Ascaris lumbricoides*.VO_2_ max was influenced by sex and age but not helminth infection and intensity, *P*. *falciparum* parasitaemia or environmental parameters. Two stools and urine samples were investigated but neither Hb nor anthropometry was measured.

Hürlimann and colleagues [14] investigated 219 children aged 8–14 years before and after deworming. Only one stool, urine and blood sample was collected. *S*. *mansoni*, *S*. *haematobium*, *Plasmodium* spp, STHs (mainly hookworm), *Giardia*, *Entamoeba*, anaemia, anthropometry, socio economic status, physical fitness and cognitive function were tested. Physical fitness was tested with a battery of three tests including the 20mSRT. Unexpectedly, the VO_2_ max was slightly lower after treatment although this difference was not significant. In line with the study of Bustinduy and colleagues [11], the study found a significant association between fitness and nutritional deficiencies [14].

The study that compares best with the present study is from Kenya by Samuels and co-workers [13]. A total of 822 children aged 7–8 years were investigated for morbidity related to *S*. *mansoni* taking three stool samples. They also determined Hb, anthropometry and PedsQL, and had data on STHs and *P*. *falciparum* parasitaemia. They did not find any significant associations between anthropometric measurements (stunting and wasting), 20mSRT or PedsQL outcomes and *S*. *mansoni* in the multivariable analyses.

A few earlier studies have investigated the effect of *S*. *mansoni* infection on physical fitness but most of these have focused on adults and used different tools for measuring fitness. While one study could not find a relationship between presence or absence of *S*. *mansoni* and productivity in Sudanese can-cutters using a stationary bicycle ergometer [30], El Karim and co-workers [31] found a relationship between both intensity and duration of *S*. *mansoni* infection and work performance in the same study population using the same physiological tests. In Zimbabwe there was an effect of the presence of infection on physical performance in sugar cane cutters using the Harvard Step Test [32], and bonus earnings as a proxy for productivity was also affected by infection with *S*. *mansoni* in cane cutters in Tanzania [33]. In one of the few studies in children, the effects of schistosomiasis on a running test in South African children were inconclusive [34].

The US findings of enlarged livers, enlarged spleens, enlarged livers and spleens, and increased portal vein diameter in the present study were a bit lower compared to the results reported in an earlier study in school and pre-school children aged 3–13 years in the same part of Tanzania [9], but overall mean Hb levels in the two studies were comparable. Prevalence of *S*. *mansoni* was much higher in the present study which is expected as the larger trial deliberately selected villages with high prevalence. In contrast to this, geometric mean intensities among positives were comparable in the two studies. So, the slightly lower percentages of enlarged organs cannot be explained by lower levels of prevalence and intensity of *S*. *mansoni* infections. We would have expected to find a positive relationship between US detected hepatomegaly and high *S*. *mansoni* intensity as found earlier in the study area [9] as well as in a study in Kenya with comparable levels of heavy infections and hepatomegaly [13], but maybe our study did not have enough power to find a possible relationship. It was, however, not surprising to find so few with PPF as it is not uncommon to find schoolchildren with enlarged livers in the absence of US detectable fibrosis. While a peak in hepatomegaly follows the peak in *S*. *mansoni* infection intensity, the peak in PPF is at a much higher age as described by Wilson and colleagues [10].

The lack of relationship between hepatosplenomegaly and *S*. *mansoni* intensity is, on the other hand, expected as this was not found earlier [9]. In spite of 16% children with heavy infections in the present study, we only found two children with very heavy infections (>2000 epg); an intensity which have been shown to be the only level where there is a relationship between hepatosplenomegaly and *S*. *mansoni* infection [7]. Furthermore, it is known that chronic exposure to malaria results in hepatosplenomegaly in schoolchildren and that infection with both malaria and *S*. *mansoni* has been found associated with hepatosplenomegaly while either infection alone has not [10].

We found no association between the presence of *S*. *mansoni* infection or intensity and any of the anthropometric parameters despite evidence from earlier studies that parasitic diseases may result in stunting [7,35]. There was also no relationship between *S*. *mansoni* and Hb/anaemia and this is in accordance with some studies from communities on the Kenyan, Tanzanian and Ugandan part of Lake Victoria shorelines [7,24,36]. On the other hand Samuels and co-workers [13] found a relationship between *S*. *mansoni* infection and anaemia controlling for *P*. *falciparum* infection in Kenya. Other factors such as inadequate dietary intake of iron, haemoglobinopathies and other infectious diseases like *S*. *haematobium*, *P*. *falciparum* and STHs play a role for growth retardation and Hb status and anaemia [35]. Unfortunately, none of these parameters were investigated in our study and especially the lack of information on *P*. *falciparum* and hookworm infections, which are very common in the area [23,24], is a limitation as these infections will influence the immune responses of the children and thereby also influence the level of schistosome related pathology.

This study had both strengths and limitations. As it is obviously difficult to show causality in cross-sectional studies, it is strictly speaking associations that we detect. On the other hand, the multivariable linear regression analysis is the strongest analysis we have for the available data, and a range of very different parameters were controlled for in the analyses. Although the Kato-Katz thick smear technique is not very sensitive we have improved the sensitivity by taking one stool sample on three consecutive days and made duplicate smears of each sample. Loss to follow-up in this cohort during the four years of intervention is unavoidable, so sample size will decrease over the years making the baseline data the strongest data for a study on associations.

In conclusion, of the different *S*. *mansoni* related morbidity parameters measured, only the emotional dimension of quality of life was significantly associated with *S*. *mansoni* infection. With only three studies investigating the effect of schistosome infection on quality of life using the PedsQL, and with conflicting results, it is not obvious whether the PedsQL could be a useful tool to measure schistosome related morbidity. To shed more light on this, more in depth studies in different endemic areas are needed in order to refine the tool so it focus more on aspects of quality of life that may be affected by schistosome infections.

## Supporting Information

S1 STROBE checklist (PDF)

S1 Trial protocol (PDF)

S1 Data (XLS)
