# Supplementary material for: Infection with Schistosoma mansoni has an Effect on Quality of Life, but not on Physical Fitness in Schoolchildren in Mwanza Region, North-Western Tanzania: A Cross-Sectional Study
Source: PLoS Negl Trop Dis. 2016 Dec 27;10(12):e0005257. doi: 10.1371/journal.pntd.0005257 (PMC5222294; doi:10.1371/journal.pntd.0005257)
Supplement: S1 Trial protocol — (PDF) [file pntd.0005257.s002.pdf]

Evaluation of Subtle Morbidity due to *Schistosoma mansoni* in endemic areas of Mwanza region, Tanzania, where prevalence is 25% or above.

By

Annette Olsen (PI) [1], Pascal Magnussen [1], Safari Kinung´hi [2]

[1] DBL-Centre for Health Research and Development, Faculty of Life Sciences, University of Copenhagen, Thorvaldsensvej 57, DK-1871 Frederiksberg C, Copenhagen, Denmark
AO: Phone: +45 3533 1403, Fax: +45 3533 1433, aol@life.ku.dk
PM: Phone: +45 3533 1436, Fax: +45 3533 1433, pma@life.ku.dk
www.dbl.life.ku.dk

[2] National Institute for Medical Research, Mwanza Research Centre, Isamilo Road, P. O. Box 1462, Mwanza, Tanzania
SK: Phone: +255 028 2500399, Cell: +255 784 318096, e-mail: kinunghi_csm@hotmail.com; skinunghi@nimr.or.tz

April 2010
Amended protocol August 2011

Abstract
This study will evaluate subtle morbidity related to infections with *Schistosoma mansoni* and will be part of a bigger study on Gaining and Sustaining Control of schistosomiasis. The study will be conducted in an area of Mwanza Region in Tanzania, where prevalence of *S. mansoni* is 25% or more. The present study will involve the two most extreme of the six study arms of the control study. The primary outcomes of interest will be measured in a cohort of children (7-8 years of age at the initiation of the intervention study) in Years 1, 3 and 5 prior to that year's MDA. A total of 800 children, namely 100 children from 4 randomly selected communities/villages from each subtle morbidity study arm will be enrolled for the 4 years of intervention and the fifth year of follow-up testing. The prevalence and intensity of *S. mansoni* will be determined in Years 1, 3 and 5 as one stool sample on three consecutive days will be examined by duplicate Kato-Katz. Date of birth will be ascertained for all participating children. The following indicators of subtle morbidity will be measured: height and weight, hemoglobin (capillary blood), physical fitness (shuttle run test), symptoms and well-being (PedsQL), Test of Everyday Attention for Children (TEA-Ch), and abdominal ultrasound (only in Years 1 and 5). All children will be treated with mebendazole and an effort will be made to evaluate malaria treatments in the communities. The study will be managed by the National Institute for Medical Research (NIMR), Mwanza, Tanzania and DBL-Centre for Health Research and Development (DBL), Copenhagen, Denmark. The study will take five years to complete and the total budget is 60.000 USD.

Research goal
The overall goal of this study of subtle morbidity is to provide information to policy- and decision-makers about the impact of MDA with praziquantel in schistosomiasis-endemic areas.

Methodology
<u>Study area and population</u>
Investigations of subtle morbidity will be conducted as part of the study on Gaining and Sustaining Control in high prevalence communities. The study area is described in the control proposal (RFP-SCORE-1-3/1.5 2009/Tanzania 2010). The subtle morbidity study will involve two of the six study arms in the main study, which represent the most and least intensive intervention arms. The testing of subtle morbidity will be conducted in Years 1, 3 and 5 prior to that year's annual MDA.

<u>Study design and sample size</u>
The study population for the Gaining and Sustaining control study will involve repeated cross-sectionals of a random selection of children aged between 9-12 years, first year students (aged ≤ 8 years) and adults (aged 20-55 years). For the subtle morbidity studies, the primary outcomes of interest will be measured in a cohort of 7-8 year old children selected at the initiation of the intervention study. 100 children from 4 communities/villages in each subtle morbidity study arm (1 and 2, see figure on next page) will be enrolled in the subtle morbidity studies for the 4 years of intervention and the fifth year of follow-up testing (5 years total). Thus, 800 children will be followed, but testing will only be conducted in Years 1, 3, and 5, prior to that year's annual MDA (arrows on the figure).

Villages for inclusion in this study will be a random sample of the villages in the 2 study arms, but they can be selected from among a restricted group of no less than 10 of the 25 villages in each arm, which could be chosen, for example, for convenience.

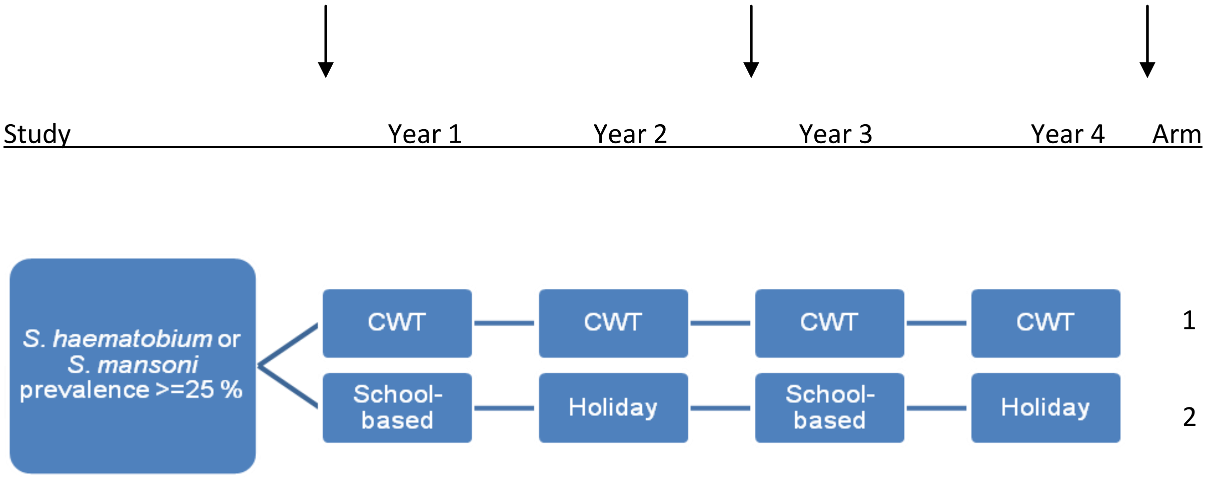

<u>Selection of children for the cohorts</u>

If more than 100 children are potentially eligible for the study, a random selection of 100 children will be performed. If less is available, a random number of 9 years old children will be selected.

<u>Basic data to be collected on all children</u>

All children in the cohort will be tested in Years 1, 3, and 5 for *S. mansoni.* One stool on 3 consecutive days will be examined using Kato-Katz, 2 slides per stool. Date of birth will be ascertained for all participating children – possibly using immunization cards.

<u>Core indicators</u>

All measures below will be conducted in Years 1, 3, and 5, except for ultrasound, which will be done at the beginning and end of the study (Years 1 and 5).

1. Height and weight.
2. Hemoglobin from capillary blood using the Hemocue
3. Physical fitness based on the shuttle run protocol.
4. Symptoms and well being based on PedsQL. This instrument will need to be translated and validated prior to being used.
5. ~~Test of Everyday Attention for Children using the TEA-Ch tool (Clarke et al., 2008).~~
6. Abdominal ultrasound in Years 1 and 5 (Richter et al., 2001).

Training of study staff

Staff should be trained on all procedures and SOPs will be provided by the SCORE Secretariat following development and agreement by those involved. The SCORE Secretariat will explore options for training on the shuttle run.

Other parasitic infections

All included children will be treated with mebendazole against soil-transmitted helminths. An effort will be made to evaluate malaria treatments in the communities; approaches to doing this may involve key informants, market surveys, or asking parents or children about whether malaria treatment has occurred and which drugs have been used.

Mid-course Evaluation

The research team are committed to participate in a mid-course evaluation after Year 2, which could result in changes in study design or funding, for example, changing interventions in certain study arms or redirecting funds to answer critical questions arising in the course of the funded research.

Treatment of infected individuals

All individuals who are tested and found to be positive for schistosomiasis will be treated, either as part of the study or on an individual basis if they are no longer in school. While this may affect some parameters of interest, we are ethically bound to offer treatment to individuals who are known to be infected.

Collection, distribution, and sharing of samples and data for use by a range of research groups

The research team will facilitate the collection, analysis and sharing of samples and data with other SCORE researchers.

Project management

The study will be managed by the National Institute for Medical Research (NIMR), Mwanza Research Centre, Mwanza, Tanzania and DBL-Centre for Health Research and Development (DBL), Faculty of Life Sciences, University of Copenhagen, Denmark.

Annette Olsen (AO) will be principal investigator and overall responsible for the financial management of the project. Pascal Magnussen (PM) will assist in the supervision of the project and give technical assistance in the field. Safari Kinung'hi (SK) will be overall responsible for the project in the field and train, supervise and support the field staff. SK will be responsible for data collection and quality control. SK conducted his PhD study in Mwanza region and is very familiar with the people, the health staff and health facilities in the area.

Activities and Timelines (in red, combined with the activities and timelines for the Gaining and Sustaining Control proposal)

	2010				2011				2012				2013				2014				2015			
Quarter	1	2	3	4	1	2	3	4	1	2	3	4	1	2	3	4	1	2	3	4	1	2	3	4
Submission for ethical clearance	X																							
Identification of study communities		X																						
Informing of health officials		X																						
Purchase of materials			X																					
Screening of villages for inclusion					X	X																		
Training of teachers and CHWs							X																	
Randomization						X																		
Community mobilization							X																	
Census							X																	
Baseline parasitological survey							X	X																
Training of study staff						X	X																	
Morbidity assessments								X								X								X
Delivery of treatment								X				X				X				X				
Follow-up parasitological surveys											X	X			X	X			X	X				
Final follow-up parasitological survey																							X	X
Laboratory work					X	X	X	X	X	X	X	X	X	X	X	X	X	X	X	X	X	X	X	X
Collection of cost data											X													
Health education								X				X				X				X				
Annual SCORE meeting						X				X				X				X				X		
Mid-course evaluation														X										
Data analysis and report writing																					X	X	X	X

Budget summary (see detailed budget in the appendix)

	**Year 1**	**Year 2**	**Year 3**	**Year 4**	**Year 5**	**Total**
**Personnel**	12,900		9,150		11,400	33,450
**Field costs**	4,440		4,440		4,440	13,320
**Equipment**	3,710		0		0	3,710
**Consumables**	2,543		2,060		2,060	6,663
**Overhead to Tanz (5%)**	1,180		783		895	2,857
**Overall total**	**24,773**		**16,433**		**18,795**	**60,000**

Dissemination of results

The results of this study will be disseminated to the study villages through public meetings. The results will be published in peer reviewed journals with all investigators as authors. The research team will work cooperatively with the SCORE Secretariat and other SCORE-funded investigators. If appropriate, the team will publish jointly with other teams making similar or related studies.

References

Clarke, SE, Jukes , MCH, Njagi JK, Khasakhala L, Cundill, B, Otido J, Crudder, C, Estambale BBA, Brooker S (2008). Effect of intermittent preventive treatment of malaria on health and education in schoolchildren: a cluster-randomized, double-blind, placebo-controlled trial. *Lancet*, 372; 127-138.

Richter, J, Domingues, ALC, Barata, CH, Prata, AR, Lambertucci, JR (2001). Report of the second satellite symposium on ultrasound in schistosomiasis. *Mem Inst Oswaldo Cruz*, 96 Suppl; 151-156.
